# Introduction to a Special Issue on Innovations and Controversies in Brain Imaging of Pain: Methods and Interpretations

**DOI:** 10.1097/PR9.0000000000000771

**Published:** 2019-08-07

**Authors:** Karen D. Davis

**Affiliations:** a Department of Surgery, Institute of Medical Science, University of Toronto, Toronto, ON, Canada; b Krembil Brain Institute, Krembil Research Institute, Toronto Western Hospital, University Health Network, Toronto, ON, Canada

**Keywords:** Brain imaging, fMRI, PET, Chronic pain, Machine learning

## Abstract

This special issue comprised 14 articles from leaders in the field, that provide opinions and reviews of concepts that are central to the next generation of pain imaging studies. Topics include cutting-edge technologies and approaches that are at the forefront of such studies, as well as developments toward biomarkers of pain and clinical applications that bring us closer to harnessing understanding of pains and its modulation to offer better options to those suffering from pain.

Why do we feel pain? Why does pain sometimes become chronic? How can we treat pain effectivity in all those that suffer? These are some of the fundamental questions posed by those who study and treat pain, and those living with pain. The opportunities afforded by 21st century technological innovations in human brain imaging hold great promise to answer these questions.

Until the 20th century, it was not possible to peer into the inner workings of the human brain without opening the skull. Thus, we could only infer the brain mechanisms underlying human pain through animal models and from studies in humans based on postmortem evaluation, intraoperative observations, and the impact of injuries and natural lesions (eg, stroke). That all changed when electroencephalography (EEG) was developed in the early-mid 20th century followed by the introduction of the ^133^Xe inhalation method, positron emission tomography and single photon emission tomography in the 1970s and 1980s,^24^ advances in magnetoencephalography (MEG) in the 1980s and 1990s, and then the introduction of functional magnetic resonance imaging (MRI) and structural MRI in the early 1990s.^12,24^ These approaches were first applied to the study of pain by the pioneering works emanating from the labs of Bushnell, Davis, Apkarian, Ploner, Tracey, and others.^1,2,8,9,23,26^ This early era of imaging pain was followed by an explosion of brain imaging studies (predominantly using functional MRI) throughout the 2000s that provided insight into mechanisms of acute and chronic pain.^1,8,20^ Then at the beginning of the 2010s, creative ideas advanced through technical improvement made possible more sophisticated approaches and new strategies. This reinvigorated EEG and MEG and launched the current era dominated by exciting topics as dynamic imaging and connectivity, multivariate (and multivoxel) pattern analysis, and machine learning for basic discovery and prediction of pain chronicity and treatment outcomes.^13–15,18,25,27^

This special issue comprised 14 articles from leaders in the field, that provide opinions and reviews of concepts that are central to the next generation of pain imaging studies, cutting-edge technologies and approaches that are at the forefront of such studies, developments toward biomarkers of pain, and clinical applications that bring us closer to harnessing understanding of pains and its modulation to offer better options to those suffering from pain.

Although this special issue focuses on human studies, we felt it important to open the issue with a review of recent imaging studies of pain models in animals. DaSilva and Seminowicz^5^ present data from animal imaging studies with a focus on delineating plasticity in the transition from acute to chronic pain. Animal models continue to provide important insights that cannot always be derived through human studies and thus add to the pipeline of concepts to be tested in subsequent human studies.

Next, as an entrée to articles on human brain imaging studies of pain, Davis and Cheng^6^ (reviewed by an independent editor) discuss their concept of functional and structural set points in the brain and consider how they represent state pain vs trait pain. They raise fundamental issues pertaining to the fluctuations in chronic pain across different timescales, an important consideration for neuroimagers that can inform study design and interpretation of brain imaging findings. Necka et al.^22^ then explore the complex methodological issues related to pain dynamics and approaches to image these fluctuations in brain structure and function underlying acute and chronic pain conditions related to state and trait pain measures.

The special issue then presents an article dedicated to the brainstem, an area that contains key structures underlying pain modulation and ascending nociceptive pathways information from orofacial regions. Most brain imaging studies of pain focus on the cortex because there are major technical challenges inherent in imaging small brainstem structures that are susceptible to noise related to respiration and cardiac pulsations. In Napadow et al.,^21^ review strategies are discussed that can be used to facilitate studies of acute and chronic orofacial pain while mitigating these technical obstacles.

Two articles demonstrate how noninvasive brain stimulation can be used as a tool to reveal fundamental brain mechanisms related to pain. Hohn et al.^10^ discuss neural oscillations that are associated with different types of pain and describe how transcranial alternating current stimulation can be used to modulate or entrain oscillations. Then, Weissman-Fogel and Granovsky^29^ provide a scoping review on the use of transcranial magnetic stimulation to create a virtual lesion within pain-associated brain areas. Both transcranial alternating current stimulation and transcranial magnetic stimulation could be further developed for therapeutic pain management purposes.

Four articles then address different ways to study chronic pain using brain imaging. Holmes et al.^11^ consider structural MRI findings and machine learning to understand pain subtypes (migraine and irritable bowel syndrome). Moayedi and Hodaie^19^ review advances in MRI diffusion imaging to evaluate white matter and its utility to evaluate trigeminal nerve and brain structural abnormalities associated with orofacial pain (temporomandibular disorder and trigeminal neuralgia) and to predict treatment outcomes. Loggia et al.^16^ review the utility of arterial spin labeling to assess cerebral blood flow related to experimental pain, postsurgical, chronic pain, and treatment-related effects. Finally, DaSilva et al.^4^ provide an overview of the positron emission tomography imaging and how it has advanced our understanding of opiodergic systems related to acute and chronic pain modulation, placebo, and nocebo effects.

A multimodal approach to pain management includes nonpharmacological, nonsurgical therapies. Two articles in this special issue focus on the development of such approaches. Zeidan et al.^30^ present the findings from brain imaging studies of mindfulness-based mental training that provide insight into neural mechanisms underlying pain relief. The article by Cunningham et al.^3^ then discusses how psychological therapies for pain such as cognitive behavioural therapy can induce brain plasticity related to pain relief.

Finally, this special issue concludes with articles by van der Miesen et al.^28^ and Mackey et al.^17^ who review the current state of the field in brain imaging–based biomarker development, assessment, and application to acute and chronic pain. This provides insight into the crucial step needed to translate knowledge from imaging studies of acute and chronic pain toward a personalized approached to pain management, a step that requires development and evaluation of biomarkers of pain within a neuroethical framework as set out by a task force of the International Association for the Study of Pain.^7^

## Disclosures

The author has no conflict of interest to declare.
